# Ersteinschätzung HNO-spezifischer Notfälle – eine Machbarkeitsstudie

**DOI:** 10.1007/s00106-024-01434-x

**Published:** 2024-02-22

**Authors:** Eva Krafft, Stefan Kaulitz, Johannes Voelker, Jonas Engert, Björn Spahn, Rudolf Hagen, Kristen Rak

**Affiliations:** Klinik und Poliklinik für Hals‑, Nasen- und Ohrenkrankheiten, plastische und ästhetische Operationen, Universitätsklinikum Würzburg, Josef-Schneider-Straße 11, 97080 Würzburg, Deutschland

**Keywords:** Software-Tools, Krankenhaus-Notfallambulanz, Triage, Bedarfseinschätzung, Symptombeurteilung, Software tools, Emergency service, hospital, Triage, Needs assessment, Symptom assessment

## Abstract

In Deutschland besteht seit einigen Jahren ein erhöhtes Aufkommen von Patientenfällen in der Notaufnahme, bei welchen es sich oft nicht um Notfälle für das Krankenhaus handelt. Zur Verbesserung der Triage und Lenkung der Patientenströme wurde das Triage-System SmED (Strukturierte medizinische Ersteinschätzung in Deutschland) entwickelt. Dieses zertifizierte Medizinprodukt soll sowohl die Dringlichkeit von Alltagsbeschwerden als auch den adäquaten medizinischen Versorgungsbedarf zielgerichtet, schneller und sicherer einschätzen. Mit Handlungsvorschlägen unterstützt es medizinisches Fachpersonal bei der Triage, wobei die Endverantwortung beim Fachpersonal selbst bleibt. Für das Fachgebiet der HNO-Heilkunde erfolgte anhand von 9 spezifischen Notfällen eine inhaltliche Überprüfung hinsichtlich der Plausibilität und der Patientensicherheit im Kopf-Hals-Bereich. Die Fälle wurden durch 9 HNO-Fachärzte simuliert und anhand des Medizinprodukts SmED durch medizinisches Fachpersonal und Studierende der Medizin triagiert, indem eine Versorgungsebene sowie ein -Versorgungszeitpunkt (Dringlichkeit) zugeordnet wurde. Die Mehrzahl der Fälle wurde korrekt zugeordnet. Das Ersteinschätzungssystem SmED stellt eine gute Möglichkeit dar, dringliche Krankheitsbilder der Hals‑, Nasen- und Ohrenheilkunde einzuschätzen. Langfristiges Ziel der Ersteinschätzung ist es, Kapazitäten von Ambulanzen zukünftig zu entlasten. Um dies zu erreichen und Patientenwartezeiten zu verkürzen, wäre es notwendig, zügig auf die HNO-Fachdisziplin zu verweisen. Es gilt daher sicherzustellen, dass über das Online-Tool Patienten an einen HNO-Bereitschaftsdienst weitergeleitet werden.

## Abstract (en)

In Germany, an increased volume of patient cases in the emergency department has been observed for several years, many of which do not represent hospital emergencies. To improve the triage and management of patient flows, the Structured Initial Medical Assessment (*Strukturierte medizinische Ersteinschätzung in Deutschland*, SmED) triage system was developed. This certified medical product is intended to assess both the urgency of everyday complaints and their adequate medical care in a targeted, faster, and safer manner. It supports medical professionals in triage by suggesting courses of action, whereby the ultimate responsibility remains with the professionals themselves. For otorhinolaryngology, a content review was carried out with regard to plausibility and patient safety in the head and neck region on the basis of nine specific emergencies. The cases were simulated by nine otorhinolaryngologists and triaged on the basis of the SmED by medical staff and medical students by assigning a care level and timepoint (urgency). The majority of cases were correctly assigned. The SmED initial assessment system is a good tool for assessing urgent clinical pictures in ear, nose, and throat (ENT) medicine. The long-term goal of the initial assessment is to relieve the capacities of outpatient departments. In order to achieve this and to shorten patient waiting times, it would be necessary to refer patients quickly to otorhinolaryngology. It is therefore necessary to ensure that patients are referred to an ENT on-call service via the online tool.

Viele Patientenfälle in den Notaufnahmen stellen keinen Notfall für das Krankenhaus dar. Mit dem medizinisch zertifizierten Triage-System SmED (Strukturierte medizinische Ersteinschätzung in Deutschland), einem neuen Online-Tool, sollen Patientenfälle vorab triagiert und der richtigen Versorgungsebene zeitgerecht zugeordnet werden. Im HNO-Fachbereich gibt es hierzu bislang noch keine Erfahrungen hinsichtlich der fachlichen Deutlichkeit/Korrektheit und der Patientensicherheit. In dieser Arbeit werden anhand von 9 HNO-spezifischen Notfällen die korrekte Dringlichkeit sowie die adäquate Zuordnung evaluiert.

## Ziel des Online-Tools

Seit Jahren steigt die Zahl der Patienten^1^, die sich bei Auftreten auch nur leichterer Symptome direkt an eine Notaufnahme wenden, da sie die Dringlichkeit einer direkten Behandlung, nicht richtig einschätzen oder nicht wissen, an welchen Vertragsarzt sie sich wenden können. Den Notfallambulanzen werden dadurch wichtige Kapazitäten genommen, was bei einem hohen Aufkommen von Fallzahlen zur Überlastung des Systems führen kann [1–3].

Der kassenärztliche Bereitschaftsdienst bietet seit 2012 die bundesweit einheitliche Telefonnummer 116117 an. Über diese können Patienten, bei denen es sich um keinen akuten Notfall handelt, außerhalb der Sprechstundenzeiten anrufen und sich hinsichtlich der Versorgungsebene und des -Zeitpunkts beraten lassen. Der Weg über die 116117 hat zum Ziel, den Patienten den Zugang zum ambulanten Sektor der Notfallversorgung zu erleichtern und diesen bundesweit zu vereinheitlichen [4]. Hier soll insbesondere eine medizinische Ersteinschätzung stattfinden. In einer der ersten Auswertung der strukturierten medizinischen Ersteinschätzung über die 116117 zeigte sich ein breites Spektrum von Behandlungsanlässen. Nach einer Analyse des Zentralinstituts für die kassenärztliche Versorgung (Zi) meldeten sich die Patienten mit 105 verschiedenen Behandlungsanlässen. Demnach riefen knapp eine Millionen Patienten von Mai 2020 bis Mai 2021 die Servicenummer des Systems der Kassenärztlichen Vereinigung (KV) an. Etwa 110.000 Anfragen seien auf COVID-19-Informationen zurückzuführen gewesen. Mehr als 850.000 Anrufende meldeten sich mit anderen Beschwerden wie Rücken- (81.546) oder Bauchschmerzen. Anderen Beschwerdebildern waren 752.000 Anrufe zuzuordnen [5].

Im Auftrag des Zi wurde ein Instrument zur Ersteinschätzung mit dem deutschen Handelsnamen *Strukturierte medizinische Ersteinschätzung in Deutschland* (SmED) entwickelt. SmED basiert auf dem Schweizer System SMASS des Berner Unternehmens in4medicine AG. Mit dem Ersteinschätzungssystem SmED, welches als Medizinprodukt zertifiziert ist, sollen sowohl die Dringlichkeit von Alltagsbeschwerden als auch der adäquate medizinische Versorgungsbedarf zielgerichtet, schneller und sicherer eingeschätzt werden. Das Ersteinschätzungssystem SmED wird seit dem 01.01.2020 in allen 116117 Servicezentralen eingesetzt, um medizinisches Fachpersonal am Telefon der Servicezentralen des ärztlichen Bereitschaftsdiensts unter der Nummer 116117 bei der systematischen Abfrage von Symptomen in Verbindung mit allgemeinen und symptomspezifischen Risikofaktoren (z. B. Alter, Geschlecht, Begleitbeschwerden) zu unterstützen [6]. Seit dem Beginn wurden etwa 5 Mio. Beratungen mit dem System durchgeführt. Resultate der strukturierten Ersteinschätzung mit dem Ersteinschätzungssystem SmED sind stets die beiden Dimensionen Zeitpunkt (Time-to-Treat), zu welchem eine ärztliche Versorgung erfolgen sollte (sofort, heute oder in den nächsten Tagen) und Ort der Versorgung (Point-of-Care), wie z. B. Rettungsdienst, Notaufnahme, Bereitschaftsdienstpraxis [7].

Da der klassische Notfall i. d. R. offensichtlich ist und grundsätzlich durch eine Blickdiagnose registriert wird, ist das Ziel des Ersteinschätzungssystems SmED, auch nicht offensichtliche abwendbar gefährliche Verläufe zu identifizieren und von harmlosen Alltagsbeschwerden zu unterscheiden. Das Ersteinschätzungssystem SmED wurde konzipiert, um Handlungsvorschläge anzubieten, die Endverantwortung liegt jedoch beim medizinischen Fachpersonal selbst [8].

In der Notfallmedizin werden international bereits strukturierte Ersteinschätzungsinstrumente bei der Patientenpriorisierung in der Notfallversorgung angewandt [9]. Ziele der Triage sind ein möglichst geringer Ressourcenverbrauch, Praktikabilität, Validität, gute Reliabilität, Patientensicherheit und eine von persönlichen Erfahrungen unabhängige Sicht [10]. Das Manchester-Triage-System (MTS) sowie der Emergency Severity Index (ESI) sind die meistgenutzten Triage-Systeme in Deutschland [9]. Das MTS basiert auf einem symptombasierten Ansatz [11]. Es ist eine Face-to-Face-Triage und unterliegt einer 5‑stelligen Skalierung. Auch telefonische Triage-Instrumente wie das Telephone Triage and Advice (TTA) oder das Swiss Medical Assessment System (SMASS) kommen in der Notfallversorgung zum Einsatz. Als Ergebnis der Ersteinschätzung wird der Patient in eine von 4 Dringlichkeitsstufen (Systemversion des Ersteinschätzungssystems SmED von 2021: Notaufnahme, ärztliche Beurteilung sofort, medizinische Beurteilung heute, medizinische Beurteilung später) eingeteilt. Gleichzeitig wird ein Versorgungsdispositiv festgelegt, d. h. beispielsweise Empfehlung zur Selbstbehandlung oder eines bestimmten Versorgungssettings.

Im Jahr 2017 wurden in einer durch das Zi in Auftrag gegebenen Studie die genannten Triage-Systeme anhand von Gütekriterien wie Patientensicherheit untersucht. Bei allen Systemen bestand noch Adaptionsbedarf. So lag in vielen Fällen eine Unter- oder Übertriage, besonders bei Subgruppen wie Kindern, vor. Hinsichtlich der Praktikabilität bestanden kleinere Unterschiede, v. a. bezüglich der Möglichkeit, die Instrumente gezielt weiterzuentwickeln. Hier wurde dem SMASS das höchste Potenzial zugeschrieben [12].

## Fragestellung

Generell ergibt sich die Notwendigkeit, eine inhaltliche Plausibilitätsprüfung sowie eine Zuordnung der einzelnen Dringlichkeits-Beratungsanlass-Kombinationen zu den verfügbaren Versorgungssettings durchzuführen [9]. Hierzu soll nach der Studie in einer weiteren Ebene die Funktionalität der strukturierten Ersteinschätzung im Rahmen von Pilotprojekten geprüft werden.

Symptome und Krankheitsbilder, die dem Fachgebiet der Hals‑, Nasen- und Ohrenheilkunde (HNO) angehören, werden ebenfalls im Regelwerk des Ersteinschätzungssystems SMED erfasst. Im Kopf-Hals-Bereich bestehen oft komplexe Zusammenhänge, die aufgrund der anatomischen Nähe schnell zu vital bedrohlichen Komplikationen führen können.

Für das Fachgebiet der HNO-Heilkunde besteht hier Bedarf einer inhaltlichen Überprüfung hinsichtlich der Plausibilität und der Patientensicherheit. In der vorliegenden Arbeit sollte das Regelwerk dieses neuen digitalen Systems hinsichtlich seiner fachlichen Korrektheit anhand von medizinischem Fachpersonal überprüft werden.

## Studiendesign und -methode

Für die vorliegende Studie wurde vom Zi ein Testzugang des Ersteinschätzungssystems SmED zur Simulation bestimmter Krankheitsbilder zur Verfügung gestellt. Die Erlaubnis zur Nutzung des Ersteinschätzungssystems SmED für diesen Test wurde vom EU-Importeur (Health Care Quality Systems GmbH, HCQS, Göttingen, Deutschland) und dem Zi gewährt.

**Abb. 1:**
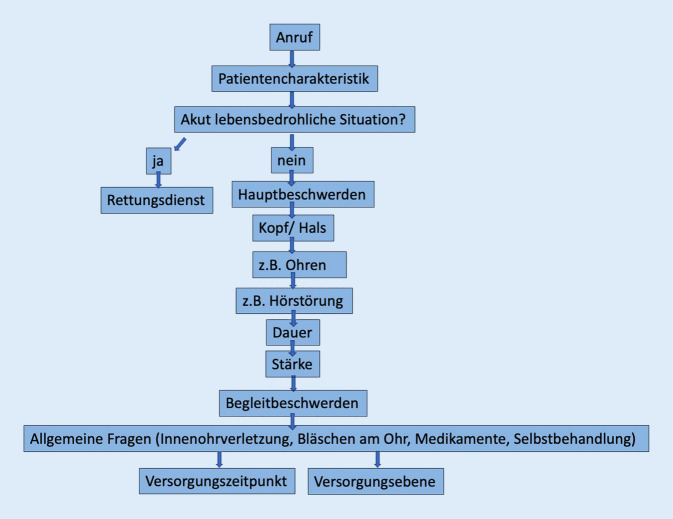
Flow-Chart: Das Ersteinschätzungssystem SmED (Strukturierte medizinische Ersteinschätzung in Deutschland) am Beispiel „Hörsturz“

Das Gespräch wurde mit einer digitalen Videokonferenz-Software (ZOOM) aufgezeichnet. Zielparameter waren die Gesprächsdauer bis zur Empfehlung und die Zuordnung zur vorher definierten Versorgungsebene und dem Versorgungszeitpunkt (Abb. 1 und 2).

**Abb. 2:**
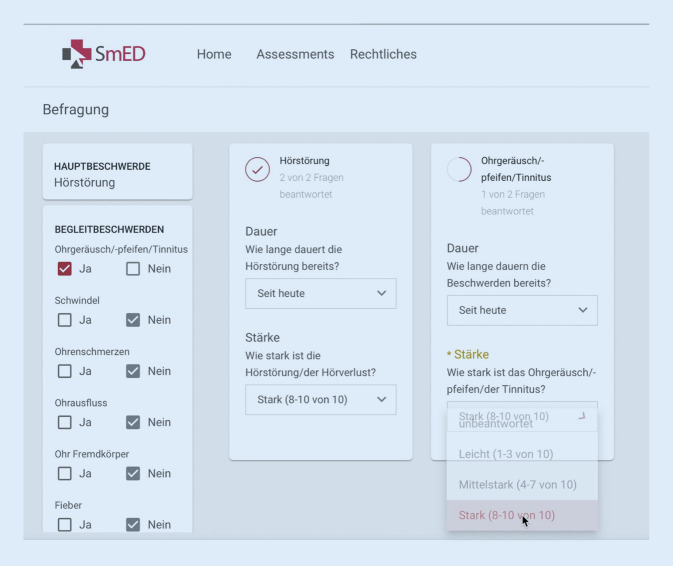
Ausschnitt der Algorithmus-Maske des Ersteinschätzungssystems SmED (Strukturierte medizinische Ersteinschätzung in Deutschland [13]), Beispiel „Hörsturz“

### HNO-Notfälle

**Abb. 3:**
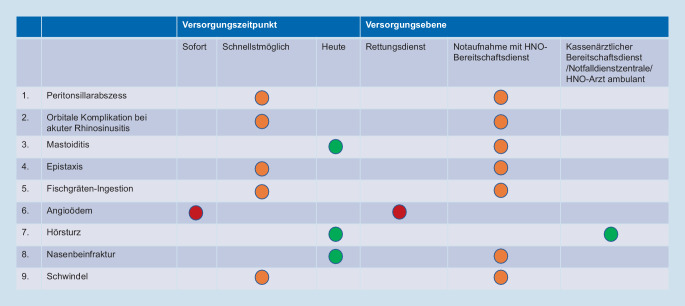
Zuordnung der HNO-Notfälle hinsichtlich Versorgungszeitpunkt und -ebene

Die Notfälle wurden anhand ihrer klinischen Häufigkeit und hohen Dringlichkeit ausgewählt. Die Abstufungen des Vorstellungszeitpunkts für hohe Dringlichkeiten im Ersteinschätzungssystem SmED waren „sofort“, „schnellstmögliche Vorstellung“ und „heute“. Es wurden HNO-Notfälle ausgewählt, welche mindestens einem der Versorgungszeitpunkte entsprachen (Abb. 3).

Für jeden Fall wurde eine adäquate Dringlichkeitsstufe und Versorgungsebene definiert. Es wurde davon ausgegangen, dass der Begriff ärztlicher Bereitschaftsdienst auch einen fachspezifischen Bereitschaftsdienst in einer Klinik einschließt, also beispielsweise eine HNO-Poliklinik (Tab. 1).

**Tab. 1:** Beschreibung und Erläuterung der Patientennotfälle 1–9

	Fall 1: Peritonsillarabszess	Fall 2: Hörsturz	Fall 3: akute Rhinosinusitis mit orbitaler Komplikation	Fall 4: Epistaxis	Fall 5: Schwindel	Fall 6: Angioödem	Fall 7: Fremdkörper (Fischgräte)	Fall 8: Mastoiditis	Fall 9: Nasenbeinfraktur
Beschreibung	18- bis 28-jähriger Patient	50- bis 65-jährige Patientin	4- bis 49-jähriger Patient	66- bis 80-jähriger Patient	66- bis 80-jährige Patientin	50- bis 65-jähriger Patient	1- bis 49-jähriger Patient	Kind 4–8 Jahre	Bis 70-jährige Patientin
	Seit einer Woche Odynophagie	Seit heute starke einseitige Hörstörung mit Tinnitus	Gesichtsschmerzen seit 2–10 Tagen	Nasenbluten seit 15 min trotz Abdrücken anhaltend	Nicht lageabhängiger Drehschwindel seit heute	Seit heute Atemnot	Fremdkörper heute ingestiert	Otorrhö	Initiale Epistaxis
	Kurzzeitige Besserung auf orales Antibiotikum	Kein Schwindel oder Otalgie	Nasenatmungsbehinderung mit periorbitaler Gesichtsschwellung	Mehr als 3 Handvoll Blut bis jetzt verloren	Hörminderung mit Tinnitus, Übelkeit und Erbrechen	Lokalisation der Schwellung: Mund/Wange/Lippe/Zunge	Schluckbeschwerden, Speichelfluss	Schwellung retroaurikulär	Stolpersturz unter Antikoagulation
	Nun Aphagie		Keine NNH-Op. innerhalb der letzten 14 Tage	Es kam zu mehr als 5‑mal Nasenbluten in den vergangenen 2 Tagen			Keine Atemnot	Trinkt weniger als sonst	
				Blutverdünnende Medikamente, bekannter Hypertonus					
Erläuterung	Der Simulierende soll sich hier als ein junger Erwachsener vorstellen und schildern, dass er seit einer Woche Halsweh habe und nun eine akute Dysphagie bestehe	Hier soll ein älterer Patient dargestellt werden, welcher unter einer schmerzlosen einseitigen Hörminderung im Sinne eines Hörsturzes leidet. Durch das Fehlen von Otalgie oder Vertigo soll eine Abgrenzung zu einem akuten entzündlichen Geschehen erfolgen	In diesem Fall kann der Simulierende entscheiden, ob er einen jugendlichen oder Erwachsenen darstellen möchte. Zeitlich soll hier eine akute und therapierefraktäre Erkrankung dargestellt werden	Im Fall der Epistaxis handelt es sich um einen hilflosen älteren Patienten, welcher bereits versucht hat durch Kompression die Blutung zu stoppen. Hier sollte die Red Flag die unstillbare akute Blutung sein	Beim hier beschriebenen Schwindel könnte es sich beispielsweise um eine M.-Menière-Attacke handeln. Auf die periphere Genese soll der Drehschwindel und die Hörminderung deuten	Der Notfall des Angioödems soll keinen Aufschub dulden, es soll eine Schwellung der oberen Atemwege simuliert werden	In diesem Fall wird ein erwachsener Mann dargestellt, welcher nach dem Verzehr oben genannter Struktur ein akutes Fremdkörpergefühl im Hals verspürt. Durch das Fehlen von Atemnot soll die Achtsamkeit auf die Ingestion gelegt werden	Der Simulierende stellt sich am Telefon als besorgtes Elternteil vor, das neben der Verschlechterung des Allgemeinzustands des Kindes auch eine retroaurikuläre Schwellung festgestellt hat	Der Fall umfasst die Situation einer älteren Dame, welche beispielsweise im häuslichen Umfeld gestürzt ist. Die initiale Epistaxis soll einerseits auf die Fraktur, andererseits wieder auf die Red Flags der aktiven Blutung aufmerksam machen

*NNH-Op.* Nasennebenhöhlen-Operation

### Fallsimulation

Das Ersteinschätzungssystem SmED wurde randomisiert in 3 Durchläufen getestet. Es wurde jeweils von 3 Assistenzärzten im ersten Weiterbildungsjahr, 3 examinierten Pflegekräften und 3 Medizinstudenten angewandt. Diese werden im Folgenden als „Dispatcher“ bezeichnet. Die Simulatoren waren jeweils 3 Fach- oder Oberärzte der Klinik, welchen randomisiert die Fälle zugeteilt wurden. Diese erhielten eine steckbriefartige Beschreibung des Krankheitsbilds (Tab. 1). Mit dieser Vorgabe sollten sie den Kasus am Telefon simulieren. Jeder Facharzt oder Oberarzt simulierte 3 verschiedene Fälle, welche jeweils von einem anderen Dispatcher bearbeitet wurden. Insgesamt ergaben sich 3 zeitliche Gruppen (Tab. 2).

**Tab. 2:** Zuordnung der Dispatcher A–I zu den jeweiligen Patientenfällen Fall 1–9 und den Simulationspatienten Oberarzt (OA)/Facharzt (FA) 1–9 in 3 Durchläufen

1. Durchlauf	Dispatcher A	Dispatcher B	Dispatcher C	2. Durchlauf	Dispatcher D	Dispatcher E	Dispatcher F	3. Durchlauf	Dispatcher G	Dispatcher H	Dispatcher I
OA/FA 1	Fall 1	Fall 9	Fall 8	OA/FA 1	Fall 1	Fall 9	Fall 8	OA/FA 1	Fall 1	Fall 9	Fall 8
OA/FA 2	Fall 2	Fall 1	Fall 9	OA/FA 2	Fall 2	Fall 1	Fall 9	OA/FA 2	Fall 2	Fall 1	Fall 9
OA/FA 3	Fall 3	Fall 2	Fall 1	OA/FA 3	Fall 3	Fall 2	Fall 1	OA/FA 3	Fall 3	Fall 2	Fall 1
OA/FA 4	Fall 4	Fall 3	Fall 2	OA/FA 4	Fall 4	Fall 3	Fall 2	OA/FA 4	Fall 4	Fall 3	Fall 2
OA/FA 5	Fall 5	Fall 4	Fall 3	OA/FA 5	Fall 5	Fall 4	Fall 3	OA/FA 5	Fall 5	Fall 4	Fall 3
OA/FA 6	Fall 6	Fall 5	Fall 4	OA/FA 6	Fall 6	Fall 5	Fall 4	OA/FA 6	Fall 6	Fall 5	Fall 4
OA/FA 7	Fall 7	Fall 6	Fall 5	OA/FA 7	Fall 7	Fall 6	Fall 5	OA/FA 7	Fall 7	Fall 6	Fall 5
OA/FA 8	Fall 8	Fall 7	Fall 6	OA/FA 8	Fall 8	Fall 7	Fall 6	OA/FA 8	Fall 8	Fall 7	Fall 6
OA/FA 9	Fall 9	Fall 8	Fall 7	OA/FA 9	Fall 9	Fall 8	Fall 7	OA/FA 9	Fall 9	Fall 8	Fall 7

**Abb. 4:**
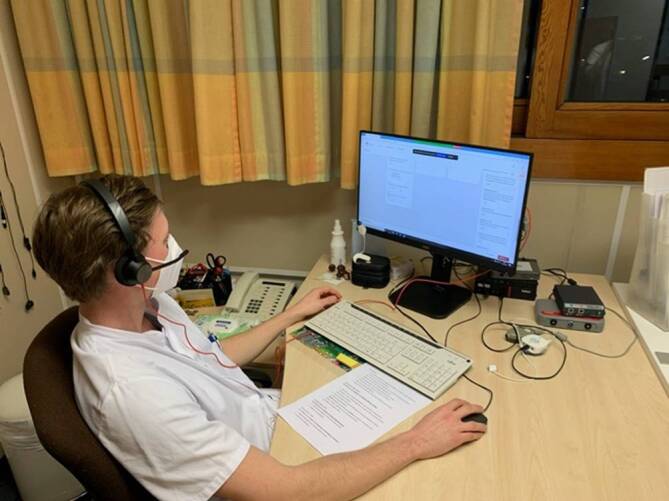
Dispatcher bei der Anwendung des Ersteinschätzungssystems SmED (Strukturierte medizinische Ersteinschätzung in Deutschland)

Für jedes Dispatcher-Patienten-Gespräch wurden maximal 10 min eingeplant. Jeder Teilnehmer verfügte während des Durchgangs über einen eigenen PC und ein eigenes Headset und wählte sich zur gegebenen Zeit in die Videokonferenz ein (Abb. 4).

Die Maske des Ersteinschätzungssystems SmED war nur für den Dispatcher sichtbar. Dispatcher und Patient konnten sich gegenseitig nicht sehen. Ein Probe-Testlauf wurde nicht durchgeführt. Die Anwendung des Ersteinschätzungssystems SmED war über die Bildschirmaufzeichnung später nachzuverfolgen und auszuwerten.

### Statistische Auswertung

Statistisch erfolgte eine deskriptive Analyse der Daten mit Microsoft Excel. In der Auswertung wurden die Mittelwerte und Standardabweichungen bestimmt. Die Berechnung der statistischen Signifikanz erfolgte mithilfe des Kruskal-Wallis-Test sowie One-Way-ANOVA.

## Ergebnisse

### Statistische Analyse aller Gruppen

Insgesamt konnten 81 Online-Telefonate ausgewertet werden. Den 9 HNO-Notfällen wurde zu 22,2 % eine sofortige, zu 45,6 % eine schnellstmögliche und zu 32,0 % eine heutige Vorstellung empfohlen. Hinsichtlich der Versorgungsebene wurde zu 55,5 % eine Vorstellung in der Notaufnahme, zu 16,0 % der Ruf des Rettungsdiensts und zu 28,3 % die Vorstellung bei einem ärztlichen Bereitschaftsdienst empfohlen.

Hinsichtlich der vordefinierten Dringlichkeitsstufe des Versorgungszeitpunkts erhielten 53 (65 ± 23 %) der Anrufer den vorher definierten Versorgungszeitpunkt zugeteilt. Hinsichtlich des Zeitpunkts der Vorstellung wurden 28 (34 ± 23 %) der Fälle über- oder untertriagiert. In Anbetracht der Versorgungsebene konnten 70 ± 21 % der Fälle der vorher definierten Ebene zugewiesen werden. In Bezug auf die Versorgungsebene waren 30 ± 21 % über- oder unterversorgt.

**Abb. 5:**
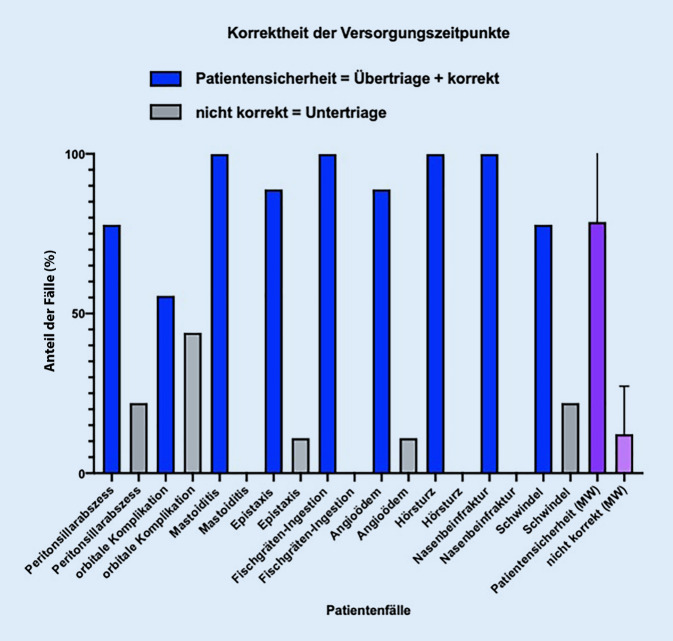
Darstellung der Korrektheit der Versorgungszeitpunkte. *MW* Mittelwert

Da nur *eine* korrekte Antwort in der klinischen Realität meist nicht vorhanden ist, wurde für die weitere Evaluation ein „range of appropriateness“ definiert. Hierzu wurde die Patientensicherheit als oberstes Ziel angesetzt, und daher wurden zu dem korrekt ermittelten Versorgungszeitpunkt und der Versorgungsebene jeweils die Patientenfälle, welche als übertriagiert oder überversorgt gewertet worden waren, addiert. Das führt dann in Bezug auf den Versorgungszeitpunkt zu einer korrekten Angabe von 87 ± 12 % und auf die Versorgungsebene von 78 ± 17 %. Hinsichtlich des Versorgungszeitpunkts bestand bei fast allen Fallsimulationen eine überwiegende Korrektheit der zeitlichen Triage (Tab. 3; Abb. 5 und 6).

**Tab. 3:** Deskriptive prozentuale Analyse aller 3 Triage-Durchläufe zu Versorgungszeitpunkt und -ebene

HNO-Notfälle (*n* = 9)	Versorgungszeitpunkt Empfehlungen					Patientensicherheit			Versorgungsebene Empfehlungen					Patientensicherheit		
	Sofort	Schnellstmöglich	Heute	Korrekt	Übertriage	*Korrekt* *+* *Übertriage*	*Nicht korrekt*	Untertriage	Rettungsdienst	Notaufnahme mit HNO-Bereitschaftsdienst	Kassenärztlicher Bereitschaftsdienst/Notfalldienstzentrale	Korrekt	Übertriage	*Korrekt + Übertriage*	*Nicht korrekt*	Untertriage
Peritonsillarabszess (*n*)	2	5	2	5	2	7	2	2	3	4	2	4	3	7	2	2
Peritonsillarabszess (%)	22	55	22	55	22	77	22	22	33	44	22	44	33	77	22	22
Orbitale Komplikation bei akuter Rhinosinusitis (*n*)	0	5	4	5	0	5	4	4	0	8	1	8	0	8	1	1
Orbitale Komplikation bei akuter Rhinosinusitis (%)	0	55	44	55	0	55	44	44	0	88	11	88	0	88	11	11
Mastoiditis (*n*)	0	3	6	6	3	9	0	0	0	7	2	7	0	7	2	2
Mastoiditis (%)	0	33	66	66	33	100	0	0	0	77	22	77	0	77	22	22
Epistaxis (*n*)	3	5	1	5	3	8	1	1	3	6	0	3	3	6	0	0
Epistaxis (%)	33	55	11	55	33	88	11	11	33	66	0	33	33	66	0	0
Fischgräteningestion (*n*)	0	9	0	9	0	9	0	0	0	8	1	8	0	8	1	1
Fischgräteningestion (%)	0	100	0	100	0	100	0	0	0	88	11	88	0	88	11	11
Angioödem (*n*)	8	1	0	8	0	8	1	1	6	2	1	6	0	6	3	3
Angioödem (%)	88	11	0	88	0	88	11	11	66	22	11	66	0	66	33	33
Hörsturz (*n*)	1	0	8	8	1	9	1	0	0	1	8	8	1	9	0	0
Hörsturz (%)	11	0	88	88	1	100	0	0	0	11	88	88	11	100	0	0
Nasenbeinfraktur (*n*)	1	6	2	2	7	9	0	0	1	7	1	7	1	8	1	1
Nasenbeinfraktur (%)	11	66	22	22	77	100	0	0	11	77	11	77	11	88	11	11
Schwindel (*n*)	2	5	2	5	2	7	2	2	2	3	4	3	2	5	4	4
Schwindel (%)	22	55	22	55	22	77	22	22	22	33	44	33	22	55	44	44
*Mittelwert (%)*	**–**					*87*	*12*	**–**						*78*	*17*	**–**
*Standardabweichung (SD)*						*0,15*	*0,15*							*0,14*	*0,15*

**Abb. 6:**
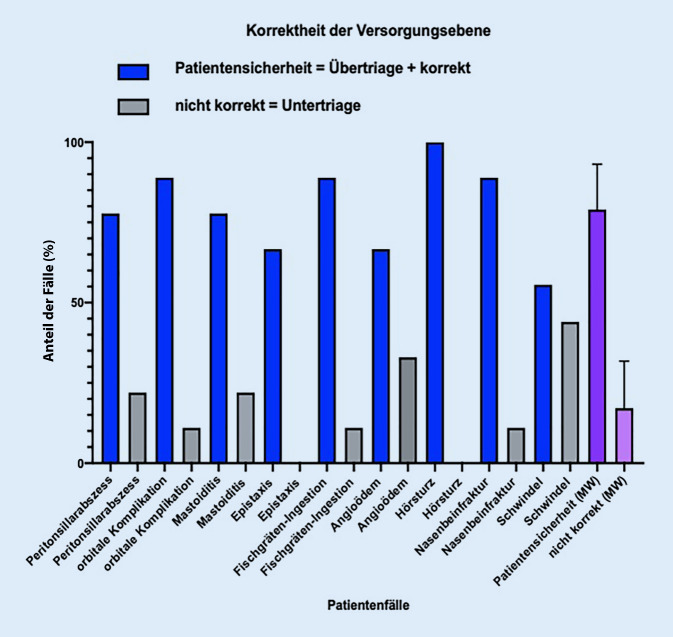
Darstellung der Korrektheit der Versorgungsebene. *MW* Mittelwert

Eine überwiegende Korrektheit der Versorgungsebene bestand auch bei der orbitalen Komplikation, der Mastoiditis, der Epistaxis, der Fremdkörperingestion, dem Angioödem, dem Hörsturz und der Nasenbeinfraktur. Beim Peritonsillarabszess bestand nur eine geringfügige Abweichung der vorgesehenen Zuordnung. Der Schwindel wurde sehr uneinheitlich zugeteilt und bot daher die größte Abweichung hinsichtlich der Versorgungsebene.

Bemerkenswerterweise wurde in 4 Fällen eine falsche Arbeitsdiagnose gestellt, aber davon in 2 Fällen dennoch eine adäquate Dringlichkeit und Versorgungsebene empfohlen. Bespielhaft wurde bei dem Krankheitsbild periorbitale Schwellung die Arbeitsdiagnose „Augenentzündung“ gestellt. Im HNO-Gebiet hätte dies die Verdachtsdiagnose „akute Sinusitis mit orbitaler Komplikation“ ergeben. Beide Arbeitsdiagnosen führten aber zur selben Dringlichkeit und Versorgungsebene.

Durchschnittlich wurden 4,4 ± 0,34 min von Beginn des Telefonats bis zur Aussprache einer Empfehlung benötigt. Nacherläuterungen der Fragen innerhalb einer Simulation bedurfte es im Mittel 1,48 ± 0,83-mal.

### Vergleich der Dispatchergruppen

**Abb. 7:**
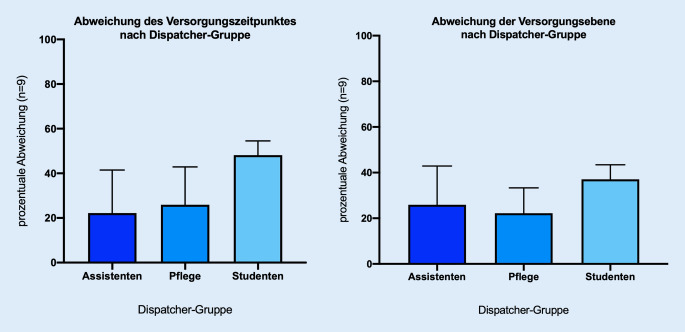
Abweichung der Korrektheit der Versorgungsempfehlung nach Dispatchergruppe

Die klinische Vorbildung der Dispatcher hatte einen Einfluss der Abwicklungszeit der Gespräche. So trauten sich die examinierten Pflegekräfte öfter, die Anrufer zu unterbrechen und in ihrer Anamneseerhebung fortzufahren, als die Medizinstudenten oder die Assistenten im ersten Weiterbildungsjahr. Die Abb. 7 zeigt hier die prozentuale Abweichung der einzelnen Dispatchergruppen von der vorgesehenen Versorgungsempfehlung. Assistenten wichen im Mittel zu 22,22 ± 15,71 % beim Versorgungszeitpunkt und zu 25,91 ± 16,97 % bei der Versorgungsebene ab. Die Abweichung bei den Pflegekräften betrug beim Versorgungszeitpunkt 25,92 ± 16,98 % und bei der Versorgungsebene 22,22 ± 11,11 %. Bei den Medizinstudenten ergab die Abweichung bei dem Versorgungszeitpunkt 48,14 ± 6,41 % und hinsichtlich der Versorgungsebene 37,03 ± 6,41 %. Die Abweichungen der vorgesehenen Empfehlungen unter den Dispatchergruppen waren nicht signifikant (Versorgungszeitpunkt *p* = 0,121; Versorgungsebene *p* = 0,424). Dennoch lässt sich tendenziell feststellen, dass die examinierten Pflegekräfte weniger Abweichungen boten. In gewisser Tendenz wichen die Medizinstudenten häufiger ab als die übrigen Gruppen (Abb. 7).

## Diskussion

### Aufbau

In der vorliegenden Arbeit wurde das Ersteinschätzungssystem SmED anhand von 9 HNO-spezifischen Krankheitsbildern auf seine Präzision der Fragen im Hinblick auf die Patientensicherheit überprüft. Dazu wurden 81 Simulationen durchgeführt und ausgewertet. In 3 Testdurchläufen wurden die spezifischen Krankheitsbilder HNO-Fachärzten und medizinischem Fachpersonal präsentiert.

Die HNO-Notfälle wurden zur verbesserten Übersichtlichkeit und Handhabbarkeit auf einen speziellen klinischen Kontext begrenzt. Dieser basierte auf den aktuellen Leitlinien zu den Patientenfällen, welche eine bestimmte Versorgung erfordern [14–22].

Für jeden Fall wurde eine adäquate Dringlichkeitsstufe und die Versorgungsebene vordefiniert. Versorgungsebene und -zeitpunkt waren nicht auf das jeweilige Erkrankungsbild hin als allgemeingültig anzusehen, sondern waren in dem jeweiligen Fall an die simulierte Anamnese und Klinik gebunden. Das bedeutet, dass es sich hier um eine allgemeine Zuordnung handelt, welche in der realen Situation auch abweichen kann, da der Disponent in der realen Anwendung auch immer die tatsächlichen Ressourcen in der Nähe des Patienten beachten muss.

Darüber hinaus muss beachtet werden, dass es immer auch einen „range of appropriateness“ gibt, also mehrere richtige Empfehlungen. Dieses wurde in die Analyse mit aufgenommen und die Patientensicherheit als oberstes Ziel angesetzt. Es wurden daher zu dem korrekt ermittelten Versorgungszeitpunkt und der Versorgungsebene jeweils die Patientenfälle, welche als übertriagiert oder überversorgt gewertet worden waren, addiert [22–25].

Die Gruppe der Fach- und Oberärzte wurde für die Simulation gewählt, da alle diese Mitarbeiter über eine Berufserfahrung von über 6 Jahre verfügten und so die Besonderheiten der Beschwerden der Patienten sehr gut aus der der eigenen klinischen Erfahrung kannten. Dies ist eine sehr wichtige Voraussetzung für eine realitätsnahe Simulation. Jedoch wurde mit ihnen keine explizite Schulung hinsichtlich der Simulation von Krankheitsbildern durchgeführt.

Als Dispatcher wurden 3 Assistenzärzte im ersten Weiterbildungsjahr und 3 examinierte Pflegekräften eingesetzt. Diese werden im Kontext der Anwendung des Ersteinschätzungssystems SmED als professionelle Anwender angesehen. Zusätzlich wurden 3 Studierende der Medizin eingesetzt, welche als Laien in Regularien des Ersteinschätzungssystems SmED benannt sind. Die Dispatcher waren vor der Anwendung nur intern geschult worden und hatten keine offizielle strukturierte Schulung erhalten [26].

Die Zuordnung der Fälle erfolgte nach einem festen Schema. Dabei wurden die 3 Fälle eines zeitlichen Blocks (1.–3. Durchlauf; Tab. 2) allen 3 Dispatchern eines Ausbildungstands vorgespielt. Zusätzlich wurden die Fälle jeweils von einem unterschiedlichen Fach- oder Oberarzt simuliert. Mit dieser Aufteilung wurde versucht, die Interrater-Variabilität und den subjektiven Bias zu reduzieren, da andere Studien gezeigt hatten, dass Unterschiede zwischen den Fachkräften auf Unterschiede in der Wahrnehmung der Aufgaben und/oder auf Unterschiede in der Fähigkeit hinweisen könnten [27] und der Umgang der gleichen Fachkräfte unterschiedlicher Versorgungsebenen mit den Patienten variieren kann [28].

Die Zuteilung der Versorgung wies eine überdurchschnittliche hohe prozentuale Patientensicherheit auf. Die meisten der Patienten sollten sich zu 45,6 % schnellstmöglich ärztlich vorstellen und zu 55,5 % eine Notaufnahme aufsuchen. Eine solche hohe Rate an Zuweisungsempfehlung zu einer Notaufnahme und dies „schnellstmöglich“ ist der Auswahl der angebotenen Notfall-Krankheitsbilder zuzuschreiben. In mehreren Studien an deutschen HNO-Kliniken konnte gezeigt werden, dass in der Versorgungsrealität ein sehr hoher Anteil an Notfällen in Klinikambulanzen im ambulanten Sektor abschließend behandelt werden kann [20–22]. Die Auswahl der angebotenen Krankheitsbilder erfolgte hier mit Blick auf die Zuverlässigkeit, schwerere Krankheitsbilder zu identifizieren.

### Vergleich mit anderer Studie

In einer Studie von van Bremen et al. wurden 1439 HNO-Notfälle retrospektiv analysiert und alle Fälle hinsichtlich Diagnose, Dringlichkeit, Therapie und Validität mithilfe des Manchester-Triage-Systems (MTS) untersucht. Hier gab es 5 Dringlichkeitsstufen (sofort – sehr dringend – dringend – normal – nicht dringend). Nach dem Triage-System sollten 0,5 % der Fälle sofort, 3,5 % sehr dringend, 44,4 % dringend, 47,3 % normal und 4,3 % nicht dringend behandelt werden. Diese mittlere Verteilung der Dringlichkeit kann auch in dieser Arbeit nachvollzogen werden. In der Studie von van Bremen konnten 2,1 % Patienten ambulant geführt werden. Bei nur 5,5 % der Fälle war eine Operation erforderlich. Die Diagnosen der stationär aufgenommenen und ambulant weiterbehandelten Patienten, die mittels MTS als „sofort“ und „sehr dringend“ behandlungsbedürftig triagiert wurden, beinhalteten wie auch in der vorliegenden Arbeit akute Blutungen, Abszesse, Quincke-Ödeme oder Schwindel. Van Bremen et al. schlussfolgern, das HNO-spezifischen Krankheitsbilder mit dem MTS sicher erkannt und korrekt triagiert werden konnten [22].

### Einstufung der Notfälle

In den vorliegenden Testläufen kam den HNO-Notfällen insgesamt eine hohe Dringlichkeit zu. Die höchste Dringlichkeit erreichte das Angioödem mit einer Schwellung der Atemwege. Bei 89 % der Fälle sollte sofort der Rettungsdienst verständigt werden. Auch bei der Epistaxis mit der unstillbaren Blutung sollte zu 33 % der Versorgungszeitpunkt sofort erfolgen und zu 56 % eine schnellstmögliche Vorstellung. Damit bestand die Fähigkeit, vitale Bedrohungen schnell einzuschätzen.

Bei der Fremdkörperingestion wurde ausnahmslos ein schnellstmöglicher Versorgungszeitpunkt zugeordnet. Zumeist sollten sich die Anrufer in die Notaufnahme begeben. Im Fall der Nasenbeinfraktur war die Schilderung der Epistaxis und der Blutverdünner die entscheidenden Faktoren für die Dringlichkeit. Handelte es sich um eine spontan wieder sistierte Epistaxis, wurde eine niedrigere Dringlichkeitsstufe ausgewählt. Daher fiel dieses Ergebnis heterogener aus.

Dem Hörsturz wurde von allen HNO-Notfällen die niedrigste Dringlichkeit zu geordnet. Gemäß der Leitlinie der Deutschen Gesellschaft für Hals-Nasen-Ohren-Heilkunde, Kopf- und Hals-Chirurgie (2014) ist der Hörsturz – auch in prognostischer Hinsicht – kein Notfall, welcher sofort therapiert werden muss. Hinsichtlich der Diagnostik und Therapie müssen das Ausmaß des Hörverlusts, etwaige Vorschäden und der subjektive Leidensdruck individuell berücksichtigt werden [20]. Hier wäre als besondere Dringlichkeit das letzthörende Ohr zu nennen. Dies wird bisher noch nicht im System berücksichtigt. Der Versorgungszeitpunkt „heute“ und die Versorgungsebene „ärztlicher Bereitschaftsdienst“ mit 89 % sind als adäquat anzusehen.

Die einzelnen Symptome des Kopf-Hals-Bereichs konnten schnell in einer Arbeitsdiagnose zusammengefasst werden. Dennoch konnten einige Krankheitsbilder nicht dem HNO-ärztlichen Bereich zugeordnet werden, wie z. B. das komplexe Krankheitsbild des Schwindels. Dieser wurde zunächst in die Neurologie eingruppiert und überschnitt sich im Gespräch mit vielen anderen Fachdisziplinen. Beispielsweise wurden aufgrund des Begleitsymptoms „Übelkeit“ auch gastrointestinale Beschwerden abgefragt. Dies führte zuweilen zu einer Verlängerung des Triage-Gesprächs. Hier betrug die Triage-Zeit in einem Drittel der Fälle genau 8,1 min. Dies ist eine deutliche Abweichung von der durchschnittlichen Triage-Zeit von genau 4,4 min (Daten nicht gezeigt).

Des Weiteren findet sich bei der Maske der Hauptbeschwerden keine Nasenatmungsbehinderung. Bei der sinugenen orbitalen Komplikation kann hier keine Konnotation zwischen der Schwellung des Auges und der Nase hergestellt werden. Hier wird das HNO-Krankheitsbild in den Bereich der Augenheilkunde weitergeleitet. In diesen Fällen wurde eine falsche Arbeitsdiagnose gestellt, was zu einer niedrigeren Rate der korrekten Aussage bezüglich des Vorstellungszeitpunkts „schnellstmöglich“ führte.

Hinsichtlich der Nacherläuterungen waren diese zumeist bei der Abfrage der Vorerkrankungen wie Immundefekte, Diabetes mellitus und Auslandsaufenthalte im Durschnitt 1,5-mal pro Gespräch nötig.

Nach Aussprache der Empfehlungen bestanden oft Fragen hinsichtlich des Transports und der Notwendigkeiten von Begleitpersonen. Dies betraf zumeist Fälle von betagten Patienten oder Jugendlichen. Hierzu gibt das Ersteinschätzungssystem SmED keine Empfehlung. Es wird in dann aber in der Realität eine Empfehlung durch das Personal der Leitstelle gegeben, welche sich an die örtlichen Begebenheiten und die Mobilität der Patienten orientiert.

Des Weiteren wäre es von Wichtigkeit, bei der Zuweisung in eine Notaufnahme in eine Ambulanz mit einer HNO-Klinik zu verweisen, da die aufgeführten Krankheitsbilder auch nur HNO-ärztlich versorgt werden können.

Bei der Systemanwendung bestanden zeitweilig Unsicherheiten der Dispatcher. So wählten sie mitunter nicht alle Qualitäten aus, die der Anrufer geäußert hatte. Bei Äußerung mehrerer Symptome wurde in einzelnen Fällen versucht, sich auf einige zu beschränken, da nicht alle in der Maske ausgewählt werden konnten. Teilweise wurden auch Symptome übersprungen, um vermeintlich Zeit zu sparen, da sich die Dispatcher von den Anrufern gedrängt fühlten. Selten wurden jeweilig Fragen ausgelassen, wie beispielsweise, ob schon einmal Paukenröhrchen gelegt wurden. Trotz des Auslassens oder Überspringen mancher Symptome erfolgte zumeist eine hohe Priorisierung und damit eine gute Patientensicherheit.

### Limitationen

Bei der vorliegenden Studie handelt es sich um eine Machbarkeitsstudie, die vor dem Hintergrund erfolgte, die grundsätzliche Patientensicherheit bezüglich des Ersteinschätzungssystems SmED in Bezug auf HNO-Fälle einzuordnen. Die methodischen Ansprüche einer klinischen Validierung wurden daher in dieser Machbarkeitsstudie nicht vollständig berücksichtigt. Folgende Punkte sind bei der Interpretation der Ergebnisse zu berücksichtigen:

Zwar verfügten die Simulationspatienten als Oberärzte und Fachärzte über ein tiefes klinisches Wissen der HNO-Krankheitsbilder, jedoch wurde mit ihnen keine explizite Schulung hinsichtlich der Simulation von Krankheitsbildern durchgeführt. Anwendungsfehler waren daher in einzelnen Fragen nicht vermeidbar, wodurch die tatsächliche Empfehlung des Systems in manchen Simulationen anders (ggf. dringlicher/weniger dringlich oder andere Versorgungsebene) hätte ausfallen können.

Für das Medizinprodukt SmED ist für die korrekte Anwendung herstellerseitig eine umfangreiche Anwenderschulung verpflichtend vorgegeben. Dies wurde im Rahmen der Studie nicht umgesetzt. Dies führte dazu, dass SmED nicht in allen Fällen in jedem Detail korrekt durchgeführt wurde; so wurden teilweise nicht alle Fragen beantwortet, sodass die tatsächliche Empfehlung des Systems in manchen Simulationen anders (ggf. dringlicher oder andere Versorgungsebene) hätte ausfallen können.

Aus pragmatischen Gründen wurde von Best-Practice-Ansätzen zur Umsetzung der Fallbeispiele sowohl hinsichtlich der Qualifikation der simulierenden Personen als auch der Strukturierung im Gespräch abgewichen. Eine Validierung der Fallbeispiele im engeren Sinne wurde für die kleine Machbarkeitsstudie als unverhältnismäßig erachtet.

Im Rahmen der Auswertungen wurde keine klassische „range of appropriateness“, also ein Bereich, der korrekt im klinischen Kontext ist, definiert. Dies erklärt die hohe Interobservervariabilität. Es wurde jedoch eine „range of appropriateness“ in Bezug auf die Patientensicherheit definiert, die zeigte, dass nur in 13,30 % (Versorgungszeitpunkt) und 17,28 % der Fälle (Versorgungsebene) die Patienten untertriagiert wurden. Dies zeigt, dass eine Einschätzung durch das System in den häufigsten Fällen zu einer sicheren Versorgung geführt hätte.

Bei den HNO-ärztlichen Notfällen handelte es sich um ausgewählte Fallbeispiele. Diese wurden zur verbesserten Übersichtlichkeit und Handhabbarkeit auf einen speziellen klinischen Kontext begrenzt und haben nicht den Anspruch, das volle Spektrum des Erkrankungsbildes abzudecken. Dementsprechend können die daraus abgeleiteten Empfehlungen nicht verallgemeinert werden.

Im Rahmen der Machbarkeitsstudie wurden auch Fälle in die Auswertung mit integriert, bei denen nicht alle Symptome abgefragt wurden bzw. Fragen übersprungen wurden. Wenn es sich um eine Validierungsstudie handeln würde, müssten sämtliche Fälle, bei denen die Fallvignette seitens des der simulierten Patienten nicht vollständig präsentiert oder seitens der Dispatcher nicht vollständig erfragt/beantwortet wurden, ausgeschlossen werden.

Die Machbarkeitsstudie bezieht sich auf eine mittlerweile veraltete Version von SmED. Somit können nicht alle Ergebnisse zweifelsfrei auf die aktuelle Version übertragen werden.

## Fazit für die Praxis

- Das Ersteinschätzungssystem SmED (Strukturierte medizinische Ersteinschätzung in Deutschland) stellt eine gute Möglichkeit dar, dringliche Krankheitsbilder der Hals‑, Nasen- und Ohrenheilkunde einzuschätzen, und gewährleistet, dass Patienten in Bezug auf die Dringlichkeit ihrer Beschwerden zuverlässig eingeordnet werden können.
- Die Mehrzahl der Fälle wurde einer korrekten Dinglichkeit der Versorgungsebene und des Versorgungszeitpunkts zugeordnet.
- Das System wurde aus der Warte der Allgemeinmedizin verfasst, daher finden sich zu einigen Begleitsymptomen andere Erkrankungsbilder, wie z. B. bei der schwindelinduzierten Übelkeit der Verweis zu gastrointestinalen Beschwerden.
- Das Ziel des Ersteinschätzungssystems SmED ist das Erreichen einer schnelleren Triage der Patienten zum korrekten Versorgungszeitpunkt und zur angemessenen Versorgungsebene.
- Langfristiges Ziel der Ersteinschätzung ist es, Kapazitäten von Notfallambulanzen und auch der HNO-Ambulanzen zukünftig zu entlasten.
- Um dies zu erreichen und Patientenwartezeiten zu verkürzen, wäre es weiterhin notwendig, zügig auf die richtige Fachdisziplin zu verweisen.
- Hinsichtlich des Fachgebiets der HNO gilt es daher sicherzustellen, dass über das Online-Tool HNO-Patienten auch in einen HNO-Bereitschaftsdienst weitergeleitet werden.

## Footnotes

1 Es sind immer beide Geschlechter gemeint. Aus Gründen der Lesbarkeit wird nachfolgend darauf verzichtet, beide zu nennen.
